# Atypical depression: state of the art and future perspectives

**DOI:** 10.3389/fpsyt.2026.1928447

**Published:** 2026-09-23

**Authors:** Gianmarco Ingrosso, Anthony J. Cleare

**Affiliations:** 1 Centre for Affective Disorders, Department of Psychological Medicine, Institute of Psychiatry, Psychology and Neuroscience, King’s College London, London, United Kingdom; 2 The National Affective Disorders Service, South London and the Maudsley NHS Foundation Trust, London, United Kingdom

**Keywords:** atypical depression, bipolar spectrum, immunometabolic depression, MAOIs, psychopathology

## Abstract

Atypical depression was first described in the 1950s, but after a surge of interest and publications in the 1980s, this construct appears to have fallen out of focus, with comparatively less literature produced over recent decades. Despite different conceptualisations proposed by different authors, atypical depression is considered by current nosographic classification systems to be characterised by mood reactivity, reversed neurovegetative symptoms such as hyperphagia or hypersomnia, marked fatigue and rejection sensitivity. Core features described in historical accounts also include prominent anxiety symptoms, reversed diurnal variation of mood, and a specific response to monoamine oxidase inhibitors (MAOIs). Many authors have reported latent or future bipolarity in subjects presenting with this depressive subtype, while others have explored a broader spectrum encompassing bipolar spectrum conditions and certain presentations of borderline personality disorder. From a pathophysiological perspective, current evidence suggests that atypical depression represents a biologically and clinically meaningful depressive phenotype, characterised by converging neuroendocrine, immuno-inflammatory, and metabolic alterations, including specific hypothalamic–pituitary–adrenal axis dysregulation and associations with obesity and type 2 diabetes. Additionally, the frequent overlap with the bipolar spectrum has implications for diagnostic formulation and treatment planning in at least a subset of patients. Notably, alongside the classically identified efficacy of MAOIs, a wide range of treatments appears also to be effective in the management of atypical depression, and emerging options like GLP-1 receptor agonists may theoretically prove preferentially efficacious in this depressive subtype.

## 1 A forgotten clinical entity?

After a period of intense research activity following its initial definition in the 1950s, and renewed interest during the 1970s and 1980s, the psychopathological construct of atypical depression appears to have been somewhat neglected by current psychiatric research. In most clinical trials, depression severity outcomes are assessed using the Montgomery–Åsberg Depression Rating Scale, MADRS (1) or the Hamilton Depression Rating Scale, HAM-D (2), which either lack dedicated items assessing atypical features or assign a score of zero to reversed neurovegetative symptoms, such as hypersomnia and increased appetite. However, other psychometric instruments exist, such as the Inventory of Depressive Symptomatology (IDS-C) scale (3), or its shorter self-report version, the Quick Inventory of Depressive Symptomatology (QIDS-SR) scale (4) that positively score atypical depressive features. Additionally, there has been limited investment in the development of new treatments or in the evaluation of currently available drugs in this depressive subtype; this may be especially relevant in the current era of precision medicine and personalised treatment.

Based on these considerations, the present review aimed to provide an updated synthesis of both seminal and contemporary evidence on atypical depression, integrating historical, psychopathological, neurobiological and therapeutic evidence to offer a comprehensive overview of the current state of the field. While previous reviews have addressed selected aspects of atypical depression, such as its historical conceptualisation or biological correlates, few have attempted to integrate these perspectives with a critical appraisal of the diagnostic validity of the construct and its treatment implications within a single, updated synthesis. Particular emphasis is placed on unresolved questions regarding the validity of current Diagnostic and Statistical Manual of Mental Disorders (DSM) diagnostic criteria, the emerging concept of immunometabolic depression, and novel therapeutic perspectives, including the rationale for investigating GLP-1 receptor agonists in atypical depression. Together, these aspects have important implications for both current clinical practice and future research, highlighting the need for improved diagnostic characterisation of atypical depression and the development of targeted therapeutic approaches.

## 2 Historical overview of the concept of atypical depression

The term “atypical depression” was first introduced towards the end of the 1950s by psychiatrists E.D. West and P.J. Dally, based at St. Thomas’s Hospital in London (5). They described a series of patients with “somewhat atypical depressive states, sometimes resembling anxiety hysteria with secondary depression”, that exhibited a specific and almost complete response to a monoamine oxidase inhibitor (MAOI), iproniazid, after other treatments had failed. Iproniazid, initially marketed as an anti-tubercular drug, had been observed to make patients more euphoric and less depressed, despite no improvement in their infectious symptoms (6).

Prominent features in the psychopathological presentation of atypical depressive patients described by West and Dally were anxiety, fatigue, and over-reactivity, which appeared to be of primary importance with respect to depression of mood. Furthermore, these subjects typically did not exhibit classic melancholic features such as weight loss, ideas of guilt, early morning awakening or a worse mood in the morning; instead, their condition tended to worsen in the evenings (i.e. reversed diurnal variation). Atypical depressive patients showed a rapid and sustained response to iproniazid, even when the drug was discontinued after a few months, and a less favourable response to treatments used in more typically endogenous forms of depressive illness, such as tricyclic antidepressants (TCAs) and electroconvulsive therapy (ECT) (5, 7).

Subsequently, Klein and Davis introduced the term “hysteroid dysphoria” to describe patients, more often women, who exhibited atypical features compared to those of melancholic depression, such as hypersomnia, hyperphagia or weight gain, marked fatigue with a sensation of “leaden paralysis” (i.e. an extreme heaviness of arms or legs), phobic-anxious traits, and a preferential response to MAOIs. These patients also displayed extreme rejection sensitivity, particularly in the context of romantic relationships, which could precipitate episodes of depressed mood, dysphoria, anger, hostility, and suicidal behaviours. In contrast to melancholic patients, they exhibited a reactivity of mood, with a rapid improvement in response to positive or desired events (8). Later descriptions remarked upon the notion of a distinct “hysterical” presentation in atypical depression, marked by traits such as “hysterical personality”, heightened anxiety, frequent somatic complaints, and a positive response to MAOIs (9), and emphasised a reversal of the typical vegetative symptoms observed in melancholic depression, with increased appetite, weight and libido (10).

At the time, then, the concept of atypical depression encompassed a range of heterogeneous conditions, defined by the exclusion of endogenous (i.e. melancholic) depression, rather than by the identification of a specific diagnostic entity (11). A significant step in the systematisation of the concept of atypical depression was made by Davidson and colleagues, who described two distinct subtypes: the “V type” (vegetative) atypical depression, characterised by increased appetite, weight, sleep or libido, and the “A type” (anxious) atypical depression, characterized by anxiety, panic, and phobic symptoms. Common features shared by the two subtypes included early onset, a higher prevalence in women, mild intensity, non-endogenicity, and minimal psychomotor change (12). In the same paper, Davidson and colleagues also highlighted three additional historical uses of the term “atypical depression”: 1) agitated psychotic patients who responded to ECT; 2) patients with residual depressive conditions such as depression secondary to schizophrenia; and 3) patients with bipolar depression exhibiting atypical vegetative symptoms that responded to MAOIs. Regarding the latter, they noted that some authors considered certain “V type” atypical depressive presentations, characterised by one or more atypical vegetative symptoms and responsiveness to MAOIs, as potentially indicative of a bipolar condition (13, 14). More specifically, Himmelhoch and colleagues introduced the term “anergic depression” to describe a form of bipolar illness characterised by reduced motor behaviour, fatigue and volitional inhibition, rather than actual mood depression or anhedonia. The latter features, when present, were considered more likely secondary to self-reproach, stemming from the attribution of volitional inhibition to laziness and indecisiveness. The condition was also associated with suicidality, generally occurring only after multiple treatment failures, and a vegetative profile including one or more of hypersomnia, weight gain or hyperphagia (14). These forms also showed a better response to the MAOI tranylcypromine compared to imipramine, which the authors attributed to its chemical similarity to amphetamine, suggesting that this characteristic made it particularly effective in cases where retardation and anergia were predominant symptoms (14–16).

Based on all these accounts, in the 1980s a group of researchers from Columbia University developed a set of specific diagnostic criteria for atypical depression (17), which were later incorporated in the fourth edition of the DSM (DSM-IV) (18). Drawing on the construct of hysteroid dysphoria, the researchers shifted the focus away from anxiety symptoms and specific personality traits noted in previous descriptions, highlighting instead mood reactivity as the defining feature. This ended up constituting criterion A of the DSM-IV definition of the atypical features specifier for Major Depressive Disorder, MDD (18). Mood reactivity refers to the ability to be at least temporarily cheered by pleasant events, in contrast to what is typically observed in melancholic presentations. Additionally, patients were required to present two or more of the following associated features: increased appetite or weight gain, hypersomnia or spending more time in bed, severe fatigue creating a sensation of leaden paralysis and rejection sensitivity (17).

The work of the Columbia research team ultimately led to a simplification of the concept of atypical depression, shifting from a view of multiple distinct forms to a single, unified clinical entity. Through a series of studies including atypical depressive patients as defined by their criteria, the Columbia group researchers were able to demonstrate that their classification, slightly different from the predominantly anxious presentations initially described by the London group, identified a distinct subgroup of patients who showed selective benefit from MAOIs compared to TCAs (11, 19). Interestingly, patients with mood reactivity but without reversed neurovegetative symptoms showed a good response to both classes of drugs (19). Overall, these findings suggested that the pathophysiology of atypical depression was distinct enough to lead to such a specific response to a particular class of antidepressants (20).

The diagnostic criteria for atypical depression, first introduced as a specifier for MDD in DSM-IV, have been retained in all subsequent editions, including the current DSM-5-TR. The specifier “with atypical features” applies to MDD and persistent depressive disorder (dysthymia) if a patient meets two criteria: (A) presence of mood reactivity, and (B) at least two of the following: hypersomnia, hyperphagia or weight gain, leaden paralysis and interpersonal rejection sensitivity (21).

## 3 Epidemiology and psychopathology of atypical depression

The presence of atypical features is common in patients diagnosed with depression. In samples of patients with MDD or dysthymia the prevalence rates range from 15% to 40%, depending on the study population (22–28). These figures are likely underestimations, given that what is considered by some to be a core feature of the syndrome, interpersonal rejection sensitivity, is considered an accessory rather than a mandatory criterion in the DSM definition, and therefore has not been specifically required in many studies (20).

Atypical depression is characterised by a higher prevalence in females, an earlier onset, younger age (12, 28, 29), a more chronic course (29), greater episode severity, and higher rates of suicide attempts (30). Additionally, comorbidity with anxiety disorders, particularly specific phobias (30), panic disorder with agoraphobia and social anxiety disorder is well documented (29). For example, a study of 243 outpatients with MDD found that patients meeting criteria for social anxiety disorder and avoidant personality disorder had a significantly higher prevalence of atypical depression (54.8%) compared to those with neither condition (31.1%) (31). The co-occurrence of social anxiety disorder appears to be particularly frequent (32, 33), echoing and intertwining with the concept of rejection sensitivity, which has been highlighted by many authors in descriptions of atypical depressive patients. The association is further strengthened by evidence of a specific efficacy of MAOIs in both atypical depression and social anxiety disorder, supporting the hypothesis of an aetiological relationship between the two syndromes (34).

Regarding other psychopathological disorders and personality traits, the presence of atypical features is also associated with overall greater lifetime psychiatric comorbidity (35), including alcohol abuse and drug dependence, dysthymia, and personality disorders of clusters B and C, excluding antisocial personality disorder (30). Additionally, in a study using a dimensional approach to personality assessment, patients with atypical depression reported higher scores on the dimension of Neuroticism, as well as on the facets of Impulsivity and Anger–Hostility (36). An association with fibromyalgia (37), somatic symptoms in general (22, 38) and body dysmorphic disorder (39) has also been documented.

Eating-related psychopathology represents another clinically relevant dimension of atypical depression, with binge eating disorder (BED) and bulimia nervosa (BN) being the most consistently reported eating disorders associated with this depressive subtype. For example, in a large community cohort, atypical depression was associated with more than twice the odds of binge eating (OR=2.2) (40), and in a study of 107 patients with atypical depression, 17.8% met lifetime criteria for BN (41).

A higher prevalence of childhood neglect or sexual abuse is another commonly reported history finding (28, 30). In this regard, Levitan proposed that early-life trauma may induce long-lasting neurobiological changes predisposing individuals to depressive presentations with reversed neurovegetative symptoms (42), potentially via altered hypothalamic-pituitary-adrenal (HPA) axis regulation (43).

Lastly, as will be later discussed in greater detail, there is evidence indicating significant associations between atypical depressive presentations and bipolar disorder I (BD-I) (30), bipolar disorder II (BD-II), seasonal depression, and the bipolar spectrum in general (40).

As mentioned above, the IDS-C scale (3) is a 30-item clinician-rated instrument that assesses general depressive symptoms and includes specific items to evaluate atypical features of depression. In a study on 1500 MDD patients using a definition of atypical depression based on IDS items, previously identified features of atypical depression such as higher prevalence in females, earlier age at onset, greater comorbidity with anxiety symptoms, and greater symptom severity were confirmed (44).

## 4 Biological correlates of atypical depression

Based on genetic and neurobiological studies, a reduction in dopaminergic transmission has been proposed as a potential mechanism underlying atypical depression (45). This hypothesis emerged during a period when aetiopathological research in psychiatry was influenced by a pharmaco-centric approach, and was supported by the observed preferential response of atypical depression to MAOIs, which exert a greater effect on dopaminergic transmission compared to other classes of antidepressants. MAOIs work by inhibiting the activity of the MAO enzyme, thereby preventing the degradation of monoamine neurotransmitters. This results in increased concentrations of these neurotransmitters in the synaptic cleft, ultimately enhancing monoaminergic transmission. Of the two isoforms of the MAO enzyme, MAO-A preferentially degrades serotonin and norepinephrine, while dopamine is metabolised by both MAO-A and MAO-B (46).

### 4.1 Genetics

Regarding genetics research, a study of 1029 female twin pairs found that individuals with recurrent depressive episodes characterised by atypical features tended to exhibit consistent symptom patterns across episodes, with higher concordance observed in monozygotic twins compared to dizygotic twins (23). Additionally, the MAOA-uVNTR genotype of the monoamine oxidase (MAO) A gene has been linked with both susceptibility to atypical depression and concentrations of homovanillic acid, a dopamine metabolite, supporting the potential role of genetic predisposition through altered dopamine turnover (47).

### 4.2 Neuroimaging

Neuroimaging studies have identified asymmetric brain activity in atypical depression, with increased activation in the right hemisphere compared to the left. This asymmetry is particularly evident in patients with early onset and chronic course (48, 49). This has been hypothesised to be related to the over-sensitivity to emotional stimuli displayed by patients with atypical features (i.e. interpersonal rejection sensitivity). Rejection sensitivity, in turn, has been linked to hyperactivity of the dorsal anterior cingulate cortex (dACC) (50). An alteration of metabolism in frontoparietal and somatosensory cortices has also been documented (51).

### 4.3 HPA axis, immunometabolic alterations and eating-related psychopathology

While melancholic depression is characterised by an increased activation of the HPA axis and a hypernoradrenergic status, evidence suggests a contrasting hypoactivity of the HPA axis in atypical depression (52–54), as confirmed by various studies (55, 56). A reduction in morning cortisol levels has been observed in atypical patients compared to non-atypical patients and controls (57). Additionally, atypical depression has been found to be associated with increased cortisol suppression following dexamethasone administration (dexamethasone suppression test, DST), in contrast to melancholic depression, where cortisol levels typically remain unsuppressed after DST (43, 58). It has been hypothesised that the hypoactivation of HPA axis in atypical depression might be specifically related to the hypoarousal symptoms typical of this condition (59, 60).

The evidence of lower cortisol and corticotropin-releasing hormone (CRH) levels has been linked to an increased inflammatory state, as evidenced by elevated levels of proinflammatory cytokines in patients with atypical depression (50). Inflammation has in fact been proved to be associated with depression, and this is especially true for the atypical subtype (50, 61). A specific immunological pattern was proposed in a study involving 105 patients with depression, 35 of whom had DSM-IV atypical features. The atypical depressive subset exhibited similar levels of interleukin 6 (IL-6) and tumour necrosis factor α (TNF-α) compared to melancholic patients, but showed decreased levels of interleukin 4 (IL-4) and increased levels of interleukin 2 (IL-2). These findings suggest that the differences between the two subtypes may be related to Th1 (IL-2) and Th2 (IL-4) cytokines rather than monocytic cytokines (IL-6 and TNF-α). The authors also hypothesised that this pattern may be related to the presence of reversed neurovegetative symptoms (62).

Integrating neuroimaging findings, psychopathological characteristics, and HPA axis alterations, Stewart and colleagues identified two distinct subsets of patients in a study involving 85 individuals with atypical depression, each characterised by specific biological correlates. The first subset included individuals with late-onset, non-chronic atypical depression, a positive response to imipramine, a left hemisphere perceptual processing bias, a hyperactive HPA axis, and a family history of less chronic and less atypical depression. These patients exhibited higher post-DST cortisol levels and, overall, more similar HPA axis function variables to those typically observed in melancholic depression. Conversely, the second subset was characterised by early onset, a more chronic course, poor response to imipramine, a relative right hemisphere perceptual processing bias, a hypoactive HPA axis, and a family history of chronic and/or atypical depression (63).

Contrasting findings about the role of HPA axis dysregulation in atypical depression have also been published. For instance, a systematic review on HPA axis alterations in atypical and non-atypical depression noted that, while some studies (particularly those using atypical depression definitions based on the presence of reversed neurovegetative symptoms) reported reduced HPA axis activity in atypical depression compared to controls, a considerable number of studies found no significant differences from controls in this regard (64). Another review also indicated that the association between decreased cortisol levels and atypical depression lacks strong support in the literature (65). Interestingly, a reduction in HPA axis activity has also been identified in other stress-related disorders such as chronic fatigue syndrome (CFS), post-traumatic stress disorder (PTSD) and fibromyalgia (54, 66). More specifically, in a study involving 15 subjects with DSM-IV atypical depression and 17 participants with CFS, both groups exhibited lower salivary cortisol levels compared to healthy controls. Furthermore, the two groups did not differ in other cortisol measures or in the presence of overlapping symptoms, such as fatigue and concentration problems (67).

Atypical depression has been also consistently linked to metabolic syndrome, a condition that represents a risk factor for cardiovascular diseases. Metabolic syndrome is defined as a combination of insulin resistance, hyperinsulinemia, dysglycaemia, dyslipidaemia, and hypertension. The diagnosis is based on the evaluation of the following parameters: waist circumference, fasting plasma glucose levels, triglycerides levels, high-density lipoprotein (HDL) levels, total cholesterol levels, and blood pressure (68). In support of the connection between metabolic syndrome and atypical depression, studies have shown that patients with obesity tend to present atypical features when depressed (69–71), and a small study on older Japanese patients with atypical and “typical” depression found a positive association between atypical depression, body mass index (BMI), and weight (72). Additionally, it has been demonstrated that overweight, obesity, and metabolic abnormalities, including leptin dysregulation, are more prevalent in atypical depression compared to melancholic or “typical” depression, and that the association between atypical depression and leptin alterations is stronger in individuals with higher adiposity (73). These findings have been confirmed by a recent systematic review, in which metabolic syndrome was found to be associated with atypical depression, but not with the melancholic or other subtypes (74). Chronic inflammation is known to play a role in the pathogenesis of both metabolic syndrome (75) and depression, for which the inflammatory hypothesis, as noted above, is one of several pathophysiological theories, strictly intertwined with neuroendocrine system alterations (76). In this regard, a study involving 111 patients with chronic melancholic depression, 122 with atypical depression, and 543 control subjects found increased levels of C-reactive protein (CRP), IL-6, TNF-α, metabolic syndrome components, BMI, waist circumference, and triglycerides in patients with atypical depression compared to both melancholic and control subjects. Additionally, atypical depression was associated with lower HDL cholesterol levels. In contrast, patients with melancholic depression exhibited higher salivary cortisol levels and a steeper diurnal slope (i.e., a greater difference between the typically higher morning levels and lower evening levels) compared to the other two groups (77). In this context, the concept of “immuno-metabolic depression” has recently been proposed, referring to a subtype of depression characterised by the clustering of atypical, energy-related symptoms (such as hypersomnia, fatigue, hyperphagia, and potentially anhedonia) together with systemic low-grade inflammation, including elevated levels of CRP, cytokines, and glycoprotein acetyls, as well as metabolic disturbances such as obesity, dyslipidaemia, and resistance to insulin and leptin (78).

As mentioned above, childhood trauma and early-life adversity are common in atypical depression, and they have more generally been recognised as key factors linking psychiatric and metabolic vulnerability through shared biological pathways. For instance, evidence from a large meta-analysis indicates that exposure to psychological stress during childhood and adolescence is associated with a persistent, albeit modest, increase in inflammatory activity across the lifespan, suggesting that early adversity may induce long-term immune system alterations that become more apparent with age (79). Building on this framework, it has been suggested that childhood trauma can become biologically embedded through dysregulation of the HPA axis and epigenetic modifications affecting stress-responsive genes, ultimately promoting a pro-inflammatory state and increasing the risk of metabolic syndrome, including obesity and insulin resistance (80). Interestingly, there is evidence that adverse childhood events contribute to the co-occurrence of metabolic and psychopathological disorders by influencing neuroendocrine, inflammatory and neurometabolic processes, indicating shared developmental pathways that may underlie both mental and cardiometabolic outcomes (81). These findings support a model in which childhood trauma may increase vulnerability to later psychopathology through inflammation-related and metabolic mechanisms, providing a plausible biological framework for understanding depressive phenotypes characterised by immunometabolic dysregulation, such as atypical depression.

The relationship between atypical depression and eating disorders also provides important insights into the biological correlates of atypical depression. As discussed in section 3, evidence suggests not only an increased prevalence of binge eating among individuals with atypical depression, but also a higher prevalence of atypical features in samples selected for binge eating, with overeating and oversleeping tending to co-occur within these groups (82). On the other hand, in a study of 345 bariatric surgery candidates, both melancholic and atypical depression were strongly associated with a diagnosis of binge eating disorder compared to participants without depressive symptoms, with the atypical subtype showing the highest odds (OR 10.11) and the greatest binge eating severity, followed by the melancholic subtype (OR 7.60), suggesting that, while particularly pronounced in atypical presentations, this association was not entirely specific to atypicality (83). Consistent with a shared neurobiological substrate, major depression with atypical features has also been associated with heightened meso-cortico-limbic reward responses to food cues, a circuitry also implicated in binge eating disorder (84). The phenomenological overlap between atypical depression and formal eating disorders, particularly BN and BED, may complicate their cross-sectional differentiation (85). Despite this potential overlap, important clinical differential elements remain. Hyperphagia occurring as part of a depressive episode is typically episodic and tends to improve alongside depressive symptoms, and atypical depression generally lacks the overvaluation of body weight and shape characteristic of BN and does not necessarily involve the subjective loss of control over eating that defines BED (21). Nonetheless, the weight gain associated with atypical depressive episodes could theoretically initiate a self-perpetuating cycle, whereby increased adiposity promotes chronic low-grade inflammation which, as discussed above, may further contribute to depressive symptomatology, either directly or through metabolic dysfunction.

## 5 Diagnostic validity of the concept of atypical depression

The definition and diagnostic validity of atypical depression have been a matter of debate since the syndrome’s first descriptions. Some authors have questioned the primacy of mood reactivity as a defining criterion (86). For instance, a study on 270 MDD patients who did not present melancholic or psychotic features, of which 31.3% met the Columbia/DSM criteria for atypical depression, assessed the validity of these criteria by analysing the presence of accessory features (criterion B) in patients with mood reactivity (criterion A). In this sample, none of the criterion B features were significantly overrepresented in non-melancholic individuals with mood reactivity, and leaden paralysis was paradoxically less common. Furthermore, criterion B symptoms were not significantly associated with covariates such as age, bipolarity, or depression severity. Notably, anxiety disorders, particularly panic disorder and social anxiety disorder, were found to be associated with greater reporting of weight gain, leaden paralysis, and rejection sensitivity. Similarly, lifetime panic disorder and social anxiety disorder were significant predictors of criterion B symptom count, highlighting their role in meeting DSM-IV atypical depression criteria in this sample (11). The results of this and other studies conducted by Parker’s research group underscore the shortcomings of the DSM definition of atypical depression. The findings suggest that the mood reactivity criterion does not distinguish atypical depression from other depressive subtypes and does not predict a higher prevalence of associated symptoms. Moreover, the accessory symptoms required by criterion B lack interdependence. Parker and colleagues conceptualise atypical depression as a form of non-melancholic disorder characterised by a predominance of anxiety symptoms over mood symptoms and a primacy of personality style (interpersonal rejection sensitivity) over mood reactivity, echoing the initial reports from the London group. Reversed vegetative symptoms such as hyperphagia and hypersomnia are viewed as adaptive homeostatic mechanisms rather than primary depressive symptoms, potentially restoring slow-wave sleep or modulating mood through increased carbohydrate intake (11, 87). This is reminiscent of hysteroid dysphoric patients described by Klein, who would resort to oversleeping and overeating when depressed due to a relationship break (8).

Posternak and Zimmerman reported similar results in a study that included 661 unipolar and bipolar depressed patients. Although some significant associations between the criterion B symptoms were identified (hyperphagia with hypersomnia and leaden paralysis; leaden paralysis and rejection sensitivity), there was no correlation between these and mood reactivity (88).

Further supporting these findings, a study compared three groups of subjects treated with desipramine: 1) patients with mood reactivity alone, 2) patients with mood reactivity plus one or more atypical associated features and 3) patients with neither mood reactivity nor atypical features. At low doses, desipramine acts as a relatively selective norepinephrine reuptake inhibitor and stimulates the release of cortisol. Patients with a “full-blown” atypical presentation (group 2) exhibited a significantly higher cortisol response compared to the other two groups (1 & 3), who did not differ in terms of cortisol response. In addition to suggesting a potentially less impaired norepinephrine system in atypical depression, the results indicated that while mood reactivity may be necessary for the diagnosis of atypical depression, the additional presence of at least one associated symptom seems to be required for a distinct biological profile (56).

Considering the limitations of the diagnostic construct of atypical depression as conceptualised by current classification systems, alternative definitions have been tested on clinical samples. In particular, Benazzi validated a simplified definition based solely on the presence of oversleeping and overeating in a sample of 202 MDD patients and 281 BD-II patients (89). The prevalence of atypical depression according to the DSM-IV criteria and the new definition was similar (42.8% and 38.7%, respectively), with both definitions showing almost identical significant associations: BD-II, female gender, younger age, earlier age of onset, Axis I comorbidities, depressive mixed states, depressive episodes lasting more than two years, and a family history of bipolar disorder. Notably, the new definition showed a significant association both with the full DSM-IV atypical depression criteria and with the specific DSM-IV symptoms that were not retained in the new definition.

The results obtained with this simpler definition were later confirmed by a factor analysis conducted on a sample of 254 MDD and 348 BD-II patients diagnosed with DSM-IV atypical depression. The analysis, which considered atypical depression validators such as female gender, early onset, BD-II diagnosis, Axis I comorbidity, and a family history of bipolarity, identified two symptom clusters: one including oversleeping, overeating, and weight gain, which was significantly associated with atypical validators, and another comprising interpersonal sensitivity and mood reactivity, which showed no significant associations, suggesting that a simpler definition focusing solely on reversed vegetative symptoms might be more accurate (90). A similar simplified definition has also been employed in other studies (22–24, 40), with two demonstrating a preferential response to MAOIs over TCAs, mirroring findings with the full DSM-IV definition (91, 92).

In contrast to these findings, an earlier study demonstrated that defining atypical depression by mood reactivity and at least one reversed neurovegetative symptom of hypersomnia, hyperphagia, or weight gain predicted non-responsiveness to imipramine. Notably, the inclusion of additional atypical features, such as atypical diurnal mood variation, leaden paralysis, and rejection sensitivity, did not further identify an imipramine non-responsive subgroup (93). It has also been suggested that considering only atypical and “typical” depressive episodes may overlook clinically relevant subgroups, as some individuals with reversed neurovegetative symptoms experience both atypical and “typical” episodes over the course of their lives. In a large stratified community sample of 8,116 individuals, including 653 subjects with lifetime major depression, Levitan and colleagues identified a so-called “fluctuating” subgroup (5.8% of depressed patients, representing approximately one third of those with reversed neurovegetative symptoms) who had experienced both atypical and “typical” episodes over their lifetime. These individuals had substantially higher rates of psychiatric comorbidity, substance misuse and mental health service utilisation than patients with only reversed neurovegetative symptoms or only typical presentations. This suggests that some of the psychopathological characteristics historically attributed to atypical depression may actually reflect the inclusion of this subgroup, thereby leading to an overestimation of the relative psychopathology of atypical depression, since individuals with only reversed neurovegetative episodes showed remarkably similar demographic and clinical characteristics to those with only “typical” episodes (24). Given the high rates of psychiatric comorbidity, substance misuse, personality pathology and mental health service utilisation associated with bipolar disorders (94, 95) and the above-discussed relationship between atypical depressive presentations and bipolarity, this subgroup may represent individuals with greater affective dysregulation and could potentially include undiagnosed bipolar disorder cases.

A recent large genetic study from the Australian Genetics of Depression Study reported interesting results that seem to support the neurobiological and clinical validity of atypical depression (96). Out of 14897 participants with depression, 3098 met criteria for atypical depression based on the presence of reversed neurovegetative symptoms (weight gain and hypersomnia) and showed an earlier age of onset, greater illness severity, higher BMI, and increased comorbidity. Atypical depression was also associated with significantly higher polygenic risk scores (PRS) for major depression, attention deficit hyperactivity disorder (ADHD), bipolar disorder and neuroticism, as well as for BMI, T2D, CRP and insulin resistance. The significant PRS associations for neuroticism and ADHD suggest the presence of neurodevelopmental and emotion regulation vulnerabilities, consistent with the mood reactivity feature of atypical depression. Interestingly, atypical depression showed significant associations with eveningness, seasonality and poorer self-reported effectiveness of Selective Serotonin Reuptake Inhibitors (SSRIs) and Serotonin-Noradrenaline Reuptake Inhibitors (SNRIs), but not TCAs, for which the association was non-significant. While sensitivity analyses adjusting for BMI attenuated most PRS associations, the chronotype PRS and poorer SSRI/SNRI response persisted, suggesting potential BMI-independent circadian mechanisms underlying atypical depression, which in turn might indicate the usefulness of circadian-targeted interventions as adjunctive treatments in this depressive subtype.

## 6 Atypical depression, the bipolar spectrum and borderline personality disorder

As previously discussed, the relationship between atypical depression and bipolar depression has been highlighted soon after the syndrome was first described.

Evidence indicates that certain forms of atypical depression, particularly those characterised by prominent reversed neurovegetative symptoms, may represent a seasonal subtype of bipolar depression (97). The presence of lethargy and reversed neurovegetative symptoms has in fact been regarded by many authors as a presentation of bipolar depression (98), and longitudinal follow-up studies have documented the development of manic or hypomanic symptoms in patients initially diagnosed with atypical depression (99). Additionally, a large study with depressed patients has found that the presence of atypical symptoms (namely hypersomnia and hyperphagia) was significantly higher in bipolar compared to unipolar patients (100). Ultimately, it appears that atypical features, although present in both unipolar and bipolar syndromes, are even more prominent in bipolar presentations compared to unipolar ones (30).

In addition to a higher frequency of atypical features in BD-I and BD-II, clinical research also supports the inclusion of atypical depression in the so-called soft-bipolar or subthreshold bipolar spectrum (39, 40, 101–103). This refers to a continuum of mood disorders that include cases exhibiting mood symptoms that are less pronounced, attenuated or subclinical, and so do not fulfil criteria for BD-I or BD-II diagnosis whilst still representing a form of bipolar illness. These include, for example, depression with brief hypomania and cyclothymic or hyperthymic temperaments. Affective temperaments refer to stable, trait-like patterns of emotional reactivity, energy, and mood regulation that are present from early life and confer vulnerability to mood disorders (104). For example, a pivotal study of 86 patients with atypical features found that as many as 72% met the broader, “soft” criteria for BD-II or related disorders. Even when applying the more conservative DSM threshold requiring hypomania lasting at least four days, 32.6% of individuals with atypical depression met the criteria for a BD-II diagnosis. Additionally, nearly 60% exhibited antecedent cyclothymic or hyperthymic temperaments, and the authors highlighted the role of such affective temperamental dysregulation and anxiety comorbidity in shaping the atypical presentation of depression, suggesting it is better classified within the bipolar spectrum than as a strictly unipolar condition (39).

Akiskal and other researchers have further expanded the concept of bipolar spectrum by proposing that certain clinical presentations of borderline personality disorder (BPD) may also fall within its boundaries. This hypothesis is primarily based on the clinical overlap observed between some forms of BPD and atypical depression (105–108), with mood instability and interpersonal rejection sensitivity representing the main shared features (109). Supporting this notion, a study on 107 patients with atypical depression found that 78% met criteria for the bipolar spectrum, and those with cyclothymic temperament were found to exhibit higher rates of lifetime comorbidities such as panic disorder, alcohol abuse, BN and borderline personality traits. Mood reactivity and interpersonal sensitivity were strongly correlated with cyclothymic traits, supporting the hypothesis of a common temperamental foundation underlying these disorders (110). Similarly, another study on 60 patients with BPD found that 81.8% had a comorbid affective disorder and specifically 27.3% had comorbid atypical depression. These patients also exhibited significantly higher levels of anxiety, global severity of psychopathological symptoms and interpersonal problems compared to those with non-atypical MDD or no depression (111).

Building on their earlier work (39), Perugi and colleagues conducted an extended study with a larger sample and additional analyses to further explore the overlap between the two diagnostic categories. In their new analysis of a sample of 107 patients with atypical depression, those meeting DSM-IV criteria for BPD showed significantly higher lifetime comorbidities, including body dysmorphic disorder, BN, narcissistic, dependent, and avoidant personality disorders, as well as cyclothymia, compared to those without BPD. BPD patients also scored higher on measures of mood reactivity, interpersonal sensitivity, functional impairment, relationship avoidance, and rejection avoidance, as well as various psychopathological factors such as anxiety, anger-hostility, and paranoid ideation. Interestingly, a logistic regression analysis indicated that cyclothymic temperament accounted for much of the relationship between atypical depression and BPD in the sample, predicting six of the nine defining DSM-IV criteria of BPD. These findings reinforce the hypothesis that trait mood lability in BPD and mood reactivity and interpersonal sensitivity in atypical depression stem from a shared cyclothymic temperamental matrix (112).

## 7 Atypical depression, seasonal affective disorder and chronic fatigue syndrome

What was previously known as seasonal affective disorder (SAD), has been reconceptualised as a specifier for affective episodes (both depressive and manic/hypomanic) since DSM-IV. A recurrent depressive episode “with seasonal pattern” is characterised by a regular temporal relationship between its onset and full remission and a specific time of the year (e.g., in the autumn or winter), with a lifetime prevalence of seasonal episodes over non-seasonal ones (18, 21). SAD has a strong familial component and occurs in two distinct types: one characterised by fall-winter depression with or without spring-summer mania or hypomania, and another by spring-summer depression with or without autumn-winter mania or hypomania (113). The autumn/winter depressive episode is often characterised by atypical symptoms such as fatigue, hypersomnia and hyperphagia with carbohydrate craving, and generally shows a good response to bright light therapy (114). The specific symptoms of SAD, particularly hypersomnia and hyperphagia, are known to be associated with hypothalamic serotonin, which fluctuates seasonally, with decreases observed during winter. The serotonin hypothesis posits that individuals with SAD have an increased susceptibility to such seasonal fluctuations in serotonin levels. This is supported by several observations, including the efficacy of drugs that enhance serotonin transmission, the craving for high-carbohydrate meals that boost brain serotonin levels, and evidence that tryptophan depletion reverses the antidepressant effects of both light therapy and the summer season in remitted patients with SAD (115). Sleep and eating patterns are known to be regulated by serotonin, and symptoms such as hypersomnia and carbohydrate craving are also common in premenstrual dysphoric disorder (PDD), a condition that shows a good response to the enhancement of serotonergic transmission. The increased carbohydrate intake observed in SAD and PDD may then represent a form of self-medicating by temporarily boosting central serotonin levels (116).

The similarities in presentation between SAD and atypical depression have prompted significant interest in exploring the relationship and potential overlap between the two syndromes. While some authors have speculated that they may represent a unitary syndrome, others (117) have emphasised the importance of clinically distinguishing between the two. Supporting the first hypothesis, a high prevalence of a seasonal pattern has been reported among atypical depressive patients (118), and the presence of atypical depressive symptoms has been shown to predict response to phototherapy in SAD (119). In contrast, a study comparing eight atypical depressive patients and 25 SAD patients found that bright light therapy was effective for SAD but not for atypical depression (114).

CFS is another condition that frequently presents with atypical depressive features. Characterised by extreme fatigue, muscle and joint pain, migraines, and concentration difficulties (120), CFS often manifests with neurovegetative symptoms such as hypersomnia, hyperphagia, and psychomotor retardation. Additionally, the hallmark symptom of CFS, extreme fatigue, bears a resemblance to the leaden paralysis commonly observed in atypical depression. These similarities have prompted speculation regarding potential overlaps between atypical depression, CFS, and SAD (60, 121).

## 8 Treatments for atypical depression

One of the most compelling pieces of evidence supporting the diagnostic validity of atypical depression is its preferential response to MAOIs over TCAs (102). As previously discussed, this holds true for the various definitions of atypical depression proposed over the past sixty years, including the original descriptions characterised by prominent anxiety and reversed neurovegetative symptoms, hysteroid dysphoric patients, and the more recent Columbia group/DSM criteria. However, some studies have reported a similar response between MAOIs and TCAs (122, 123), and it should also be noted that most historical studies have indicated a *preferential* response to MAOIs, with TCAs still improving depressive symptomatology to a lesser extent (17, 124–128). It has also been suggested that distinct patient subsets based on illness course may account for the relatively lower efficacy of TCAs in atypical depression. Specifically, patients with early-onset illness and a more chronic course appear to benefit less from TCAs compared to those with a course more closely resembling that (episodic/recurrent) of melancholic depression (129).

Phenelzine is the MAOI with the most extensive body of supporting research (17, 124–128, 130–134). Evidence also supports the use of isocarboxazid (91, 135) and tranylcypromine (14–16, 136), the latter having being specifically tested only in patients with anergic depression. The literature on the reversible MAO-A inhibitor moclobemide indicates a better tolerability profile but less satisfactory efficacy compared to other types of antidepressants, in contrast to data with irreversible MAOIs (135, 137–140). Although the irreversible MAO-B inhibitor selegiline (L-deprenyl) has been successfully used in older studies at doses higher than those routinely employed in Parkinson’s disease (141, 142), the use of transdermal selegiline has not yet been specifically tested in patients with atypical depression.

There is also evidence of a continued and specific effect of MAOIs over time. In a long-term follow-up study, patients with chronic atypical depression (i.e. duration ≥ 2 years) stabilised on imipramine or phenelzine, were randomised to 6 months of continuing their medication or switching to placebo. Recurrence rates were lowest in patients maintained on phenelzine (23%) compared to imipramine (41%), and patients who discontinued phenelzine were significantly more likely to relapse than those discontinuing imipramine, with relapse rates of 87% and 47%, respectively (143).

Clinical research has also demonstrated the efficacy of SSRIs, specifically fluoxetine (131, 144–148), sertraline (149), and escitalopram (150). Additionally, one small study in late-life/late-onset depression supports the use of venlafaxine (151), while another small open label trial documented a favourable outcome with duloxetine (152).

Medications with a prominent noradrenergic profile, such as reboxetine, have also shown efficacy (147), while findings for drugs with a putative mechanism involving dopaminergic transmission, apart from MAOIs, are mixed. Modafinil significantly improved depressive symptoms in a sample of patients with atypical depression who completed an open-label phase followed by a double-blind, placebo-controlled phase; notably, the effect was also maintained in the placebo group (153). Adjunctive aripiprazole has also been found to significantly reduce depression severity scores compared to placebo augmentation in a sample of patients with atypical depression diagnosed using the self-report version of the IDS scale (154). Conversely, augmentation therapy with bupropion failed to demonstrate significant efficacy in a trial involving DSM-IV atypical depressive patients with a history of treatment resistance, who were taking duloxetine as a baseline medication. Moreover, a higher number of atypical features was a significant predictor of non-response in this sample (155).

A recent multicentre prospective cohort study including 118 participants indicated that patients with atypical depression were significantly more likely to respond to lithium augmentation than those without atypical features, after adjustment for age, sex, baseline depression severity, and optimal lithium serum concentrations, with an adjusted hazard ratio of 2.20 (156).

In a study involving 68 patients with treatment-resistant unipolar and bipolar depression, a regimen of two intravenous ketamine infusions demonstrated efficacy in treating both melancholic and atypical depression. However, the effect sizes were moderate overall and greater in patients with melancholic depression compared to those with atypical depression (157).

Despite the original descriptions by the London group suggesting a less favourable response to ECT in atypical depressive patients, a study of 489 patients with MDD treated with ECT found that those with atypical features were nearly three times more likely to achieve remission compared to patients with “typical” features (158).

Regarding psychotherapy, evidence is more limited. A small study suggested an effect of cognitive therapy (159). Another study found comparable efficacy between cognitive therapy and phenelzine (130), while another paper reported a good effect of cognitive behavioural therapy (CBT) in reducing depressive symptoms (160). Additionally, a systematic review on CBT concluded that neither melancholic nor atypical features predicted response to this form of psychotherapy (161).

Emerging clinical evidence suggests that glucagon-like peptide-1 receptor agonists (GLP-1RAs), commonly used for the treatment of type 2 diabetes (T2D) and obesity, may also have modest but significant antidepressant effects. From a neurobiological point of view, GLP-1RAs are known to cross the blood-brain barrier and act on central nervous system targets involved in mood regulation, such as the hypothalamus and limbic structures (162), through AMPK- and GSK3β-related signalling cascades (163). Additionally, in a recent drug-target Mendelian randomisation analysis, genetically predicted higher GLP1R expression was associated with a significantly reduced risk of MDD, suggesting a potential biological basis for the protective role of GLP-1RAs in depression (164). A recent systematic review and meta-analysis of randomised controlled trials and prospective cohort studies, conducted in patients with T2D and obesity, showed that treatment with GLP-1RAs was associated with a small but statistically significant reduction in depressive symptom severity compared with placebo or other glucose-lowering agents (162). An earlier systematic review and meta-analysis of longitudinal interventional and observational studies, conducted in populations with overweight or obesity and a high prevalence of T2D, also found that treatment with GLP-1RAs was associated with a significant reduction in depression severity. Notably, when analyses were restricted to studies in patients with T2D that did not exclude subjects with depressive symptoms at baseline, GLP-1RAs showed greater antidepressant effects than control treatments. This suggests that underlying metabolic burden may influence antidepressant outcomes (163).

This preliminary evidence seems to suggest that GLP-1RAs might be particularly relevant for metabolically dysregulated depressive phenotypes, that, as discussed above, frequently present atypical features. However, it must be noted that effect sizes are generally modest, and there is substantial heterogeneity across study designs and populations in the literature on this topic, with some meta-analyses and large-scale trials finding no significant changes in depressive symptoms. Additionally, some observational and post-marketing studies have also reported an increased risk of MDD, anxiety, and suicidal behaviour associated with GLP-1RA use (165).

## 9 Limitations

The following limitations should be acknowledged. First, the available literature on atypical depression remains highly heterogeneous with regard to diagnostic definitions, which limits direct comparability across studies and may partly account for inconsistent findings regarding epidemiology, neurobiology and treatment response. Second, a proportion of the evidence reviewed derives from observational or cross-sectional studies, which limits causal interpretation. Third, despite its broad coverage, this being a narrative rather than a systematic review, it is possible that some relevant studies were missed. Finally, despite being supported by accumulating evidence, some concepts discussed in this review, including the immunometabolic model of atypical depression and the potential therapeutic role of GLP-1RA, remain hypothetical at present.

## 10 Conclusions

Although current nosographic classifications, including the DSM-5-TR and the ICD-11, have established universally used diagnostic criteria, there appear to be some questions around the definition and validity of the psychopathological concept of atypical depression. Important methodological questions remain unresolved, including whether reversed neurovegetative symptoms should be considered key manifestations, or whether mood reactivity and rejection sensitivity represent the core defining features of this depressive subtype. Notably, using current definitions, two patients with atypical depression may share only mood reactivity while differing substantially in other clinical characteristics such as reversed neurovegetative symptoms, resulting in heterogeneous presentations with potentially different treatment implications. In this regard, it is also worth mentioning that a significant proportion of patients with depression present a mixture of both melancholic and atypical symptoms (166). Despite this clinical and categorical heterogeneity, biological findings suggest that atypical depression may represent a partially distinct neurobiological phenotype characterised by specific alterations across neuroendocrine, immune, and metabolic domains. In contrast to melancholic depression, atypical depression has often been associated with relative hypoactivity of the HPA axis, including lower cortisol levels and enhanced cortisol suppression following dexamethasone administration, although findings remain heterogeneous and not universally replicated. Evidence from immunological studies indicates a pro-inflammatory profile, with alterations in cytokine signalling that may reflect specific Th1/Th2 imbalances and could be related to reversed neurovegetative symptoms. In parallel, accumulating research supports a robust association between atypical depression and metabolic dysfunction, including obesity, insulin resistance, dyslipidaemia and low-grade systemic inflammation, giving rise to the concept of an “immuno-metabolic” depressive subtype.

An important point emerging from this review is that atypical depression responds to a broader range of pharmacological treatments beyond MAOIs, although, as noted above, a recent genetic study has indicated a poorer self-perceived response to SSRIs and SNRIs in this depressive subtype (96). It should also be recognised that the reverse appears to be true, as MAOIs can have wider applicability across depressive presentations (167).

Additionally, based on the available evidence, it appears that the presence of atypical features, particularly reversed neurovegetative symptoms and marked fatigue, points towards a bipolar spectrum condition rather than a purely unipolar presentation. This distinction is especially relevant in clinical practice, as it can inform the choice of both pharmacological and non-pharmacological treatments; thus, careful longitudinal evaluation is essential in those with atypical features.

The review highlights several important gaps in our understanding of atypical depression, with direct implications for future research. First, the widely used DSM definition of atypical depression may benefit from a consensus-based reappraisal, in light of the evidence accumulated since its introduction in DSM-IV. To this end, new studies prospectively comparing DSM-based criteria with alternative definitions and dimensional approaches, and assessing symptom patterns and relevant biomarkers longitudinally, could also be informative. Second, prospective studies are needed to clarify the longitudinal relationship between atypical depression and the bipolar spectrum, including careful characterisation of lifetime depressive episode psychopathology and assessment of affective temperaments alongside other relevant variables, such as family history and other bipolarity indicators. Third, further replication is required to determine whether atypical depression includes a reproducible immunometabolic subtype. Lastly, future trials in major depression should systematically employ instruments that positively score atypical symptoms, given the implications this has for diagnostic formulation and treatment choices.
